# Integrative analysis identifies bHLH transcription factors as contributors to Parkinson’s disease risk mechanisms

**DOI:** 10.1038/s41598-021-83087-2

**Published:** 2021-02-10

**Authors:** Victoria Berge-Seidl, Lasse Pihlstrøm, Mathias Toft

**Affiliations:** 1 Department of Neurology, Oslo University Hospital, P.O. Box 4956 Nydalen, 0424 Oslo, Norway; 2 Faculty of Medicine, University of Oslo, Oslo, Norway

**Keywords:** Functional genomics, Genetic markers, Parkinson's disease

## Abstract

Genome-wide association studies (GWAS) have identified multiple genetic risk signals for Parkinson’s disease (PD), however translation into underlying biological mechanisms remains scarce. Genomic functional annotations of neurons provide new resources that may be integrated into analyses of GWAS findings. Altered transcription factor binding plays an important role in human diseases. Insight into transcriptional networks involved in PD risk mechanisms may thus improve our understanding of pathogenesis. We analysed overlap between genome-wide association signals in PD and open chromatin in neurons across multiple brain regions, finding a significant enrichment in the superior temporal cortex. The involvement of transcriptional networks was explored in neurons of the superior temporal cortex based on the location of candidate transcription factor motifs identified by two de novo motif discovery methods. Analyses were performed in parallel, both finding that PD risk variants significantly overlap with open chromatin regions harboring motifs of basic Helix-Loop-Helix (bHLH) transcription factors. Our findings show that cortical neurons are likely mediators of genetic risk for PD. The concentration of PD risk variants at sites of open chromatin targeted by members of the bHLH transcription factor family points to an involvement of these transcriptional networks in PD risk mechanisms.

## Introduction

Parkinson’s disease (PD) is a progressive neurodegenerative disorder affecting about 1% of the population above 60 years of age^1^. The cause of neuronal death is poorly understood, and this is obstructing the path toward more effective treatments. The largest-to-date genome-wide association study (GWAS) for PD identified 90 independent association signals, of which a large proportion were new compared to previous reports^2^. In spite of the last two decades’ successful identification of genetic association signals in PD and other complex diseases, translation into underlying biological mechanisms has been scarce. GWAS signals typically involve multiple variants in high linkage disequilibrium (LD), making it difficult to pinpoint the actual causal variants. In addition, most risk variants are located in the noncoding part of the genome, where the functional impact may be challenging to predict^3,4^. There is however a growing amount of epigenomic and transcriptomic data that may be integrated with GWAS findings to discover disease-relevant regulatory networks.

In previous studies, PD risk variants have been integrated with gene expression data, epigenomic annotations and functionally related gene sets to identify cell types and pathways implicated in PD pathogenesis^5–7^. Studies coupling PD risk to transcription factor binding are however scarce and there is consequently limited knowledge concerning transcriptional networks central to PD pathogenesis. Altered transcription factor binding has been shown to play an important role in human diseases^8–10^. Transcription factors bind to short and specific DNA sequences, referred to as motifs, to alter gene expression. Genetic variants may alter the binding of a transcription factor through disruption of the transcription factor recognition motif. However, the majority of variability in transcription factor-DNA binding events appear to be caused by variants outside the transcription factor recognition motif^11^. A fine-mapping study of autoimmune diseases found that predicted causal variants tend to occur near binding sites for immune related transcription factors, but only a fraction alter recognizable transcription factor binding motifs^12^.

Transcription factor binding patterns vary between cell types and may be directly assessed through chromatin immunoprecipitation sequencing (ChIP-seq)^13^. This requires one transcription factor to be tested at a time and only a fraction of transcription factor-cell type combinations has so far been assayed. Intersection of disease risk variants for 213 phenotypes with an extensive catalogue of ChIP-seq derived transcription factor binding datasets identified more than 2000 significant transcription factor-disease relationships^14^. There were however sparse findings in regard to transcription factors associated with PD, which may be explained by the small number of transcription factors assayed in neuronal cell types.

Position weight matrices, which are widely used models to describe the DNA sequence binding preferences of transcription factors, may be used to scan the genome to predict transcription factor binding sites. Importantly, transcription factors only occupy a small proportion of the genomic sequences matching to their consensus binding sites. This is because transcription factor binding is influenced by additional features such as sequence context, accessibility of chromatin and interactions among transcription factors^15,16^. Integration of genome sequence information together with cell type specific experimental data has been shown to improve the accuracy of inference of transcription factor binding^17^.

Through analysis of the overlap between Alzheimer’s disease risk variants and open chromatin sites containing specific transcription factor motifs, Tansey et al. provided evidence suggestive of specific transcriptional networks being central to Alzheimer’s disease risk mechanisms^18^. We use a similar approach integrating PD risk variants with open chromatin sites in brain, coupled with transcription factor motif analysis, to identify transcription factor networks contributing to PD risk.

## Methods

### Genomic annotations

Assay for Transposase Accessible Chromatin followed by sequencing (ATAC-seq) is a fast and sensitive method used to map genome-wide accessibility of chromatin^19^. We downloaded maps of open chromatin in neurons and non-neurons across 14 distinct brain regions of five individuals from the online database Brain Open Chromatin Atlas (BOCA). A detailed description of data generation and quality control of this dataset has been published^20^. In brief, ATAC-seq was applied to neuronal and non-neuronal nuclei isolated from frozen brain tissue by fluorescence-activated nuclear sorting. Reads were mapped to the hg19 (GRCh37) reference genome using STAR aligner v2.5.0 and peaks representing open chromatin regions (OCRs) were called using model-based Analysis of ChIP-seq (MACS) v2.1^21,22^.

The 14 analysed brain regions include different areas of neocortex, in addition to subcortical regions such as hippocampus, thalamus, amygdala, putamen and nucleus accumbens. Substantia nigra, which has a well established role in the pathogenesis of PD due to the loss of neurons in this region, was however not part of this dataset. There was to our knowledge no other data from ATAC-seq or similar assays analysing the accessibility of chromatin in human dopaminergic neurons of substantia nigra available at the time of our analysis. Pairwise intersections between genomic annotations were computed and visualized with the command line tool Intervene (version 0.6.4)^23^. Jaccard statistic was used as measure of similarity, where 0 means no overlap and 1 means full overlap.

### Genetic association signals

Genome-wide significant PD risk signals were accessed from a recent meta-analysis, which is the largest genetic study of PD to date^2^. This study, which involved the analysis of 37.7K cases, 18.6K UK Biobank proxy-cases and 1.4M controls, identified 90 independent association signals that we included in enrichment analyses. Published top-hits were accessed from Table S2 and we included the 90 association signals that were marked as having passed final filtering. We performed an additional analysis excluding the three PD risk signals located within the extended major histocompatibility complex (MHC) region (chr6: 26–34 Mb), due to the unusual LD and genetic architecture at this locus^24^.

As negative controls, we selected GWASs from non-brain related disorders that had a number of independent association signals (p-value < 5 × 10^–8^) comparable to that of the included PD meta-analysis. A GWAS of inflammatory bowel disease (IBD) (study accession GCST004131) with 94 association signals and a GWAS of peak expiratory flow (PEF) (study accession GCST007430) with 91 association signals were accessed from the GWAS catalogue^25^. As for the PD association signals, additional analyses were performed excluding one IBD risk signal and two PEF risk signals located within the extended MHC region.

### Testing for enrichment of PD risk variants in open chromatin regions

**Figure 1:**
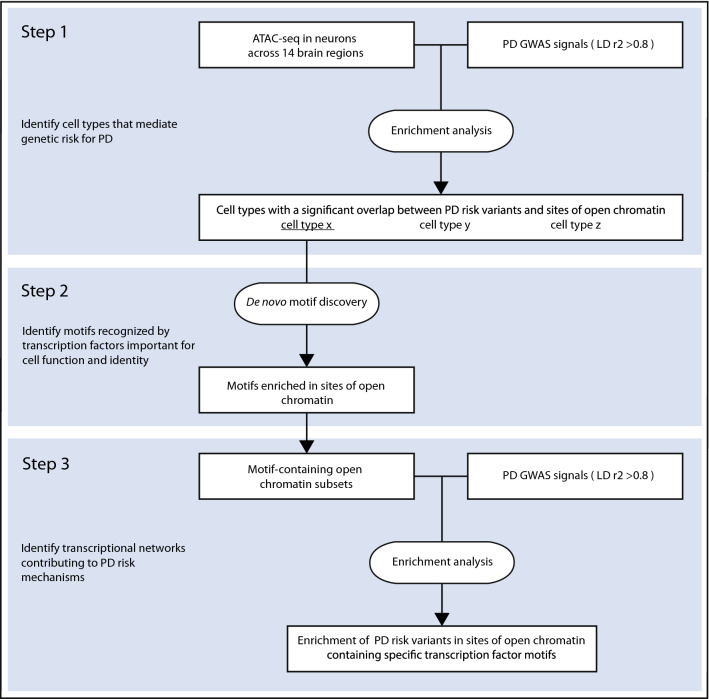
Workflow of the data analysis. PD, Parkinson’s disease; GWAS, Genome-wide association study; LD, Linkage disequilibrium.

Two methods were used to evaluate the statistical enrichment of PD risk variants and the two negative controls in OCRs defined by ATAC-seq in neurons across the 14 brain regions. We chose not to further analyse the non-neuronal cell population due to the cellular heterogeneity in this group, which contains different glial subtypes in addition to a small component of vascular cells and nucleated blood cells^26^. The workflow of our analysis is depicted in Fig. 1. First, enrichment was calculated with GoShifter, which includes genome-wide significant index variants and their LD proxies in the analysis^27^. We identified variants in LD with the index variants with the webserver Snipa (v3.3, http://www.snipa.org), using the European subset of 1000 Genomes Phase 3 v5 data and a LD threshold of r^2^ > 0.8 (Supplementary Table S1)^28^. GoShifter calculates the proportion of risk loci where at least one linked variant overlaps the tested annotation. The observed overlap is then compared to a null distribution generated by randomly shuffling the annotations within each locus, thus preserving the local genomic structure. After each shuffle, the proportion of loci overlapping annotations is calculated. We carried out 10,000 permutations to draw the null distribution.

The second method applied, GREGOR, uses a snp-matching-based method to test for enrichment^29^. The number of trait-associated signals where an index variant or one of its LD proxies overlaps a regulatory annotation is calculated, then the probability of the observed overlap of risk variants is estimated relative to expectation using a set of matched control variants. Control variants match the index variants for number of variants in LD, minor allele frequency and distance to nearest gene. European 1000 Genomes Phase 1 data is implemented in GREGOR and was used to identify LD proxies with the threshold set to r^2^ > 0.8 and a LD window at 1 Mb. The minimum number of control variants for each index variant was set at 500.

We adjusted for multiple testing by Bonferroni correction, adjusting for 14 tests in the analysis of OCRs in neurons from different brain regions and 23 tests when analysing OCRs containing specific transcription factor motifs identified by de novo motif discovery with HOMER. In enrichment analysis of OCRs harboring de novo motifs and best matched known transcription factor motifs identified with MEME-ChIP, we adjusted for 13 and 18 tests. Brain annotations that pass the significance threshold with both GoShifter (adj. p < 0.05) and GREGOR (adj. p < 0.05) are reported as significantly enriched in the text.

### De novo motif discovery and assignment to open chromatin regions

Transcription factors targeting binding motifs that are enriched in a set of regulatory regions in a cell may be regarded as candidate transcriptional regulators of that cell*.* We performed de novo motif analysis with two different softwares, HOMER v 4.10.3 and MEME-ChIP v 5.1.1, to identify motifs significantly enriched in OCRs in superior temporal cortex neurons^30,31^. HOMER identifies motifs that are enriched in the target sequences relative to GC matched background sequences. In our analysis with HOMER we used the findMotifsGenome.pl script with –size given, -mask and otherwise default settings. De novo motifs are compared against a library of known motifs in the HOMER Motif Database and all motifs in Jaspar. The identified enriched de novo motifs were assigned to OCRs using the annotatePeaks.pl script with default parameters.

MEME-ChIP performs comprehensive motif analysis of large nucleotide datasets through the combination of several motif discovery and analysis tools. Although MEME-ChIP was designed for the analysis of peak regions identified by ChIP-seq, it may also be used to identify motifs associated with genetic elements obtained by other high-throughput assays such as ATAC-seq^31^. Bedtools v2.28.2 (getfasta sub-command) was used to extract superior temporal cortex ATAC-seq peak sequences in FASTA format from the hg19 reference genome obtained from the UCSC Genome Browser^32^. The ATAC-seq peak FASTA file was used as input to analysis with the command-line version of MEME-ChIP. MEME-ChIP executes two de novo motif discovery algorithms, multiple EM for motif elicitation (MEME) and discriminative regular expression motif elicitation (DREME). MEME can find relatively long motifs, while DREME discovers short motifs up to 8 bp and is more computationally efficient. In contrast to MEME, the DREME algorithm analyses all sequences. As a default, MEME-ChIP only performs motif discovery on the central 100 bp. In our analysis the –ccut parameter was set to 0 which indicates that the full length sequences should be analysed. We used the JASPAR 2018 Core vertebrates non-redundant database for motif comparison and otherwise default settings.

The discovered motifs are grouped by similarity to each other and compared to known motifs by the Tomtom algorithm. As part of the MEME-ChIP tool set FIMO uses the most significant motif in each cluster to scan the input sequence. We used the 25 de novo motifs (most significant motif in each cluster) with lowest E-value as a basis for further analysis. All of these motifs had been identified by DREME and were thus between 6 and 8 bp long. Due to the low information content in the short 6 bp motifs, FIMO found no matches passing the default p-value threshold of 1 × 10^–4^ when scanning the large input sequence. Bedtools v 2.28.2 was used to identify ATAC-seq peak subsets containing each of the de novo motifs^32^.

The known motifs from the Jaspar database were generally longer than the de novo motifs they were matched to. Based on the assumption that the higher information content of the known motifs results in more accurate motif occurrences, we identified additional ATAC-seq peak subsets containing the best matched known motifs. Occurrences of the known motifs best matched (lowest Tomtom p-value) to the 25 most significant de novo motifs were identified with the command-line version of FIMO v 5.1.1. We used the default p-value threshold of 1 × 10^–4^ and the same Markov background model that was calculated from the input sequences by analysis with MEME-ChIP. As for the analysis of de novo motifs, Bedtools was used to identify ATAC-seq peak subsets containing each of the best matched known motifs.

Computations were performed on resources provided by UNINETT Sigma2—the National Infrastructure for High Performance Computing and Data Storage in Norway. Figures comparing multiple motifs were created with the R/Bioconductor package MotifStack v1.18.0^33^. Motif matrices provided in the HOMER Motif Database, the Jaspar database and in the output from de novo motif discovery were used as input to MotifStack.

## Results

### Similarity measures of open chromatin between brain regions and cell types

Pairwise intersections in terms of Jaccard statistics of ATAC-seq peaks representing OCRs in the different cell types show a separation between neurons and non-neurons, with the inter-region similarity being higher between the non-neurons (Supplementary Figure S1). Among the neurons, mediodorsal thalamus, putamen and nucleus accumbens differ the most from the other brain regions, while cortical regions cluster together. These results are in concordance with findings by Fullard et al., where differences were assessed between all individual samples using MDS clustering and pi1 estimates^20^.

### Enrichment of PD risk variants in neuronal open chromatin regions

PD risk variants are significantly enriched in OCRs of neurons of the superior temporal cortex (GoShifter adj. p = 0.028, GREGOR adj. p = 6.94 × 10^–05^). There is a tendency that the lowest p-values, although not significant with both enrichment tests, are in cortical regions rather than subcortical regions (Table 1). This should be viewed in relation to the high inter-region similarity between OCRs in the different cortical regions (Supplementary Figure S1). There is no evidence of an enrichment of PEF risk variants or IBD risk variants in neurons from any of the tested brain regions (Supplementary Table S2). This indicates that the enrichment of risk variants in OCRs of neurons of the superior temporal cortex is specific to PD risk variants and not to disease-associated variants in general.

**Table 1:** Enrichment of PD risk variants within open chromatin regions in neurons from different brain regions.

Cell type	GoShifter Adj. p-val (p-val)	GREGOR Adj. p-val (p-val)	No. ATAC-seq peaks
STC*	**0.028 (2.00 × 10**^**–03**^**)**	**6.94 × 10**^**–05**^** (4.96 × 10**^**–06**^**)**	76145
VLPFC	0.162 (0.012)	**2.67 × 10**^**–03**^** (1.90 × 10**^**–04**^**)**	86082
ITC	0.204 (0.015)	**4.30 × 10**^**–04**^** (3.07 × 10**^**–05**^**)**	65346
PMC	0.206 (0.015)	**3.73 × 10**^**–03**^** (2.66 × 10**^**–04**^**)**	84995
ACC	0.325 (0.023)	**7.20 × 10**^**–04**^** (5.14 × 10**^**–05**^**)**	70654
OFC	0.403 (0.029)	**3.19 × 10**^**–03**^** (2.28 × 10**^**–04**^**)**	81621
INS	0.468 (0.033)	**3.97 × 10**^**–03**^** (2.84 × 10**^**–04**^**)**	68261
DLPFC	1 (0.075)	**0.021 (1.49 × 10**^**–03**^**)**	74825
PVC	1 (0.093)	**0.014 (1.02 × 10**^**–03**^**)**	51874
NAC	1 (0.105)	0.191 (0.014)	77290
MDT	1 (0.117)	**2.97 × 10**^**–03**^** (2.12 × 10**^**–04**^**)**	69913
HIPP	1 (0.135)	**0.037 (2.66 × 10**^**–03**^**)**	80571
AMY	1 (0.151)	0.072 (5.16 × 10^–03^)	38564
PUT	1 (0.166)	0.145 (0.01)	100752

The cell type passing the significance threshold with both GoShifter and GREGOR is marked with a star and is reported in the text as significantly enriched with PD risk variants. We adjusted for multiple testing by Bonferroni correction, adjusting for 14 tests. Unadjusted p-values are provided in parenthesis. Adjusted p-val < 0.05 are written in bold. No. ATAC-seq peaks refers to the total number of peaks, representing open chromatin regions, in the analysed cell types.

PD, Parkinson’s disease; ACC, Anterior cingulate cortex; AMY, Amygdala; DLPFC, Dorsolateral prefrontal cortex; HIPP, Hippocampus; INS, Insula; ITC, Inferior temporal cortex; MDT, Mediodorsal thalamus; NAC, Nucleus Accumbens; OFC, Orbitofrontal cortex; PMC, Primary motor cortex; PUT, Putamen; PVC, Primary visual cortex; STC, Superior temporal cortex; VLPFC, Ventrolateral prefrontal cortex.

### Enrichment of PD risk variants in open chromatin regions harboring specific transcription factor motifs identified by HOMER

Candidate transcriptional regulators were assessed in OCRs in neurons of the superior temporal cortex, since this was the ATAC-seq peak set passing the significance threshold with both enrichment tests. We performed de novo motif discovery with the HOMER software and found that 22 motifs were enriched in open chromatin (Supplementary Table S3). ATAC-seq peaks were divided into 22 subsets containing each of the enriched motifs. We also created one subset with all the enriched motifs being absent (noMotif), which was intended as a negative control. HOMER compares the de novo motifs to a library of known motifs, presenting a list of the best matched known motifs based on a similarity score. The ATAC-seq peak subsets are named after the best matched known transcription factor.

When analysing HOMER motif-containing OCR subsets we found that PD risk variants were significantly enriched in OCRs harboring the de novo motif matched to the Olig2 motif (GoShifter adj. p = 0.025, GREGOR adj. p = 1.39 × 10^–03^) (Table 2). None of the other motif-containing OCR subsets were significantly enriched when both GREGOR and GoShifter were subjected to Bonferroni correction. There are however some OCR subsets that have an adjusted p-value < 0.05 with GREGOR and a nominally significant p-value with GoShifter (POL010.1_DCE_S_III, NRF1 and NFIA). There is a high degree of concordance between the highest ranked motif-containing OCR sets resulting from analysis with GoShifter and GREGOR. Also, none of the negative controls are enriched in the Olig2 OCR subset or any of the other motif-containing OCR subsets (Supplementary Table S4).

**Table 2:** Enrichment of PD risk variants within motif-containing open chromatin region sets identified with HOMER.

Motif-containing OCR sets	GoShifter Adj. p-val (p-val)	GREGOR Adj. p-val (p-val)	No. ATAC-seq peaks
Olig2*	**0.025 (1.10 × 10**^**–03**^**)**	**1.39 × 10**^**–03**^** (6.05 × 10**^**–05**^**)**	21924
POL010.1_DCE	0.064 (2.80 × 10^–03^)	**7.18 × 10**^**–05**^** (3.12 × 10**^**–06**^**)**	37574
NRF1	0.407 (0.018)	**6.26 × 10**^**–03**^** (2.72 × 10**^**–04**^**)**	7729
NFIA	0.580 (0.025)	**0.022 (9.51 × 10**^**–04**^**)**	37903
Sp2	1 (0.052)	0.147 (6.39 × 10^–03^)	7566
Egr2	1 (0.073)	0.103 (4.46 × 10^–03^)	25202
NFY	1 (0.078)	0.152 (6.63 × 10^–03^)	5806
PB0080.1_Tbp_1	1 (0.087)	0.774 (0.034)	5118
ETV2	1 (0.171)	0.113 (4.91 × 10^–03^)	10961
CTCF	1 (0.189)	1 (0.091)	6257
Mef2c	1 (0.205)	1 (0.050)	17566
PB0013.1_Eomes_1	1 (0.221)	1 (0.053)	29438
Atf1	1 (0.297)	0.331 (0.014)	5809
BORIS	1 (0.333)	1 (0.138)	6018
POL002.1_INR	1 (0.336)	1 (0.136)	34917
SPDEF	1 (0.390)	0.172 (0.007)	18884
MafF	1 (0.523)	1 (0.219)	34091
GFY	1 (0.683)	1 (0.543)	1196
Rfx5	1 (0.759)	1 (0.382)	7410
Fra1	1 (0.851)	1 (0.601)	9557
NFIL3	1 (0.901)	1 (0.723)	4431
Rfx1	1 (1)	1 (1)	3337
noMotif	1 (1)	0.542 (0.024)	1196

The motif-containing OCR set passing the significance threshold with both GoShifter and GREGOR is marked with a star and is reported in the text as significantly enriched with PD risk variants. We adjusted for multiple testing by Bonferroni correction, adjusting for 23 tests. Unadjusted p-values are provided in parenthesis. Adjusted p-val < 0.05 are written in bold. No. ATAC-seq peaks refers to the total number of peaks, representing OCRs, in the analysed motif-containing OCR sets. The total number of ATAC-seq peaks in superior temporal cortex neurons is 76145.

PD, Parkinson’s disease; OCR, Open chromatin region.

16 out of the 90 PD association signals have one or more variants in high LD located in OCRs containing the de novo motif best matched to oligodendrocyte transcription factor 2 (Olig2). Several other transcription factors are also closely matched to this de novo motif since they share very similar binding motifs (Supplementary Figure S2). This provides additional candidates potentially targeting the enriched subset of open chromatin. All candidates do however belong to the basic Helix-Loop-Helix (bHLH) transcription factor family. The noMotif OCR subset does not show a significant enrichment of PD risk variants. This subset may however not be that well suited as a negative control since it is among the smallest OCR subsets, only constituting 1,6% of the total number of OCRs.

### Enrichment of PD risk variants in open chromatin regions harboring specific transcription factor motifs identified by MEME-ChIP

Motif analysis with MEME-ChIP identified 88 de novo motifs (118 motifs clustered by similarity) to be enriched (E-value ≤ 0.05). Further analysis was limited to the 25 most significantly enriched motifs (Supplementary Table S5). Known motifs matched to the 25 most significant MEME-ChIP de novo motifs overlap several of the known motifs matched to HOMER de novo motifs (Supplementary Table S6). Transcription factors confidently matched to de novo motifs by both motif discovery tools have been described to function in neurons, such as MEF2C, SP2/SP1, NRF1 and NEUROD1/bHLH transcription factors^34–37^. We created ATAC-seq peak subsets containing each of the enriched de novo motifs. Seven de novo motifs that had no significant motif occurrences and six de novo motifs that had not been matched to any known motif (of which two had no significant motif occurrences) were excluded from further analysis. One additional subset was excluded since it was smaller than 1000 OCRs. This left 13 de novo motif-containing ATAC-seq peak subsets to be tested with enrichment analysis, of which none were significantly enriched with PD risk variants, nor with any of the negative controls (Supplementary Table S7).

Based on the assumption that known motifs matched to the short de novo motifs have a higher information content resulting in more accurate motif occurrences, ATAC-seq peaks were divided into subsets based on the location of the best matched known motifs. 19 out of the 25 de novo motifs with lowest E-value were matched to known motifs of which two were matched to the same known motif. Enrichment analysis of ATAC-seq peak subsets containing each of the 18 best matched known motifs show a significant enrichment of PD risk variants in the OCRs containing the neurogenic differentiation factor 1 (NEUROD1) motif (GoShifter adj. p = 7.20 × 10^–03^, GREGOR adj. p = 7.63 × 10^–04^) (Table 3). There is a high degree of concordance between the highest ranked motif-containing OCR sets resulting from analysis with GoShifter and GREGOR. Also, none of the negative controls are enriched in the NEUROD1 OCR subset or any of the other motif-containing OCR subsets (Supplementary Table S8). 13 out of the 90 PD association signals have one or more variants in high LD located in OCRs containing a NEUROD1 motif. NEUROD1 is a bHLH transcription factor and is interestingly among the ten known motifs best matched to the de novo motif located in the enriched ATAC-seq peak subset based on analysis with HOMER (Supplementary Figure S2).

**Table 3:** Enrichment of PD risk variants within open chromatin region sets containing known motifs identified with MEME-ChIP.

Motif-containing OCR sets	GoShifter Adj. p-val (p-val)	GREGOR Adj. p-val (p-val)	No. ATAC-seq peaks
NEUROD1*	**7.20 × 10**^**–03**^** (4.00 × 10**^**–04**^**)**	**7.63 × 10**^**–04**^** (4.24 × 10**^**–05**^**)**	12197
SP1	0.058 (3.20 × 10^–03^)	**5.79 × 10**^**–06**^** (3.21 × 10**^**–07**^**)**	19373
ZNF263	0.472 (0.026)	**2.85 × 10**^**–03**^** (1.59 × 10**^**–04**^**)**	27496
NHLH1	0.770 (0.043)	0.552 (0.031)	8288
TEAD2	1 (0.133)	0.097 (5.41 × 10^–03^)	7000
RBPJ	1 (0.137)	0.790 (0.044)	11637
KLF9	1 (0.206)	0.188 (0.010)	12364
NRF1	1 (0.254)	0.071 (3.92 × 10^–03^)	7740
SPIC	1 (0.261)	1 (0.075)	8737
SPIB	1 (0.291)	0.422 (0.023)	9300
ZIC1	1 (0.335)	0.221 (0.012)	6952
Stat5a::Stat5b	1 (0.457)	1 (0.140)	9783
ZNF384	1 (0.510)	1 (0.644)	14373
MEF2C	1 (0.557)	1 (0.279)	16923
FOSL2	1 (0.854)	1 (0.519)	9141
FOXP1	1 (0.888)	1 (0.919)	6847
TBP	1 (0.911)	1 (0.816)	5011
CREB1	1 (0.911)	1 (0.363)	2994

The motif-containing OCR set passing the significance threshold with both GoShifter and GREGOR is marked with a star and is reported in the text as significantly enriched with PD risk variants. We adjusted for multiple testing by Bonferroni correction, adjusting for 18 tests. Unadjusted p-values are provided in parenthesis. Adjusted p-val < 0.05 are written in bold. No. ATAC-seq peaks refers to the total number of peaks, representing OCRs, in the analysed motif-containing open chromatin sets. The total number of ATAC-seq peaks in superior temporal cortex neurons is 76145.

PD, Parkinson’s disease; OCR, Open chromatin region.

Enrichment testing performed with exclusion of risk signals in the extended MHC region shows similar results in all analyses to those found when including this region. OCRs in superior temporal cortex neurons, OCR subsets containing motifs linked to Olig2 and OCRs containing the NEUROD1 motif were all significantly enriched with PD risk variants also when excluding the extended MHC region. No additional OCR sets were significant with both GoShifter and GREGOR, and also no significant enrichments were found for any of the negative controls.

### Analysis of open chromatin region subsets based on two different de novo motif discovery methods point to the same transcription factor family: bHLH transcription factors

**Figure 2:**
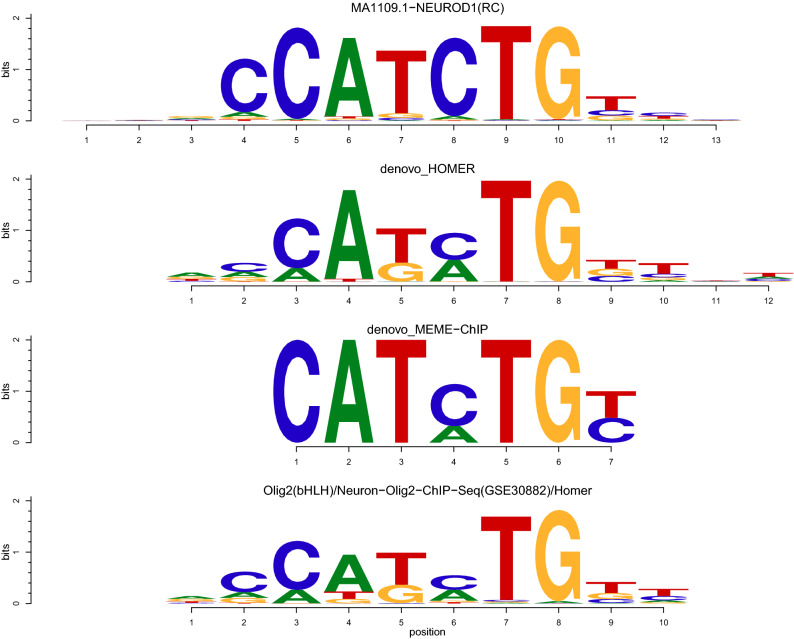
Comparison of de novo motifs matched to bHLH transcription factors. Denovo_HOMER is the de novo motif identified by HOMER, while denovo_MEME-ChIP refers to the de novo motif identified by MEME-ChIP. Olig2(bHLH)/Neuron-Olig2-ChIP-Seq (GSE30882)/Homer is the known motif best matched to denovo_HOMER and is part of the HOMER Motif Database. MA1109.1-NEUROD1 is the known motif best matched to denovo_MEME-ChIP and is part of the JASPAR 2018 Core vertebrates non-redundant database. bHLH, Basic Helix-Loop-Helix; RC, Reverse complement.

Analysis of OCR subsets based on de novo motif discovery with HOMER and MEME-ChIP both show a significant enrichment of PD risk variants in the subset targeted by bHLH transcription factors. The HOMER de novo motif matched to Olig2 and the MEME-ChIP de novo motif matched to NEUROD1 (with bHLH transcription factor motifs bhlha and TAL1::TCF3 as second and third best match) are highly similar. The similarity between these two de novo motifs, as well as between the de novo motifs and best matched known motifs, are illustrated in Fig. 2. bHLH transcription factors are known to bind to E-box motifs with the consensus sequence CANNTG, corresponding with the identified de novo motifs. In E-box motifs, the central two nucleotides and the surrounding nucleotides provide specificity of binding^34^.

The PD association signals and corresponding proxy variants that overlap the NEUROD1 OCR subset and Olig2 OCR subset are listed in Supplementary Table S9. We find high concordance between PD risk variants overlapping the two enriched motif-containing OCR subsets. 12 out of the 13 association signals and 17 out of the 20 proxy variants that locate to the NEUROD1 OCR subset are also located in the Olig2 OCR subset (Supplementary Figure S3).

## Discussion

Characterization of disease-related transcriptional networks is essential to improve our understanding of pathogenic processes and possible therapeutic targets. Identification of transcriptional networks that contribute to genetic risk mechanisms may be explored through integration of GWAS findings with epigenomic data and in silico motif analysis. This has been done in a recent study by Tansey et al., where results point to SPI1 and MEF2A/C transcriptional networks as central to Alzheimer’s disease risk mechanisms^18^. In support of these findings, variants in the proximity of both SPI1 and MEF2C have earlier been identified as significant Alzheimer’s disease risk loci^38,39^. Intriguingly, this suggests that a transcription factor may be implicated in genetic disease risk both by variants altering expression of the transcription factor itself, as well as through variants altering its binding affinity to regulatory DNA at other loci^18^.

In our study, we integrated association signals from the most recent PD GWAS with publicly available ATAC-seq data coupled with transcription factor motif analysis in an effort to identify transcriptional networks contributing to PD risk. Enrichment analysis shows that PD risk variants are concentrated at sites of open chromatin in neurons of the superior temporal cortex indicating that these cell types mediate genetic risk for PD. The finding that neurons from additional cortical regions approach the significance threshold by being significant upon multiple testing with one enrichment test and nominally significant with the other enrichment test, suggests that a broader range of cortical regions are implicated in PD risk.

The involvement of transcriptional networks was explored in neurons of the superior temporal cortex based on the location of candidate motifs identified by de novo motif discovery. Enrichment analysis shows a significant overlap between PD risk variants and OCRs harboring motifs matched to transcription factors within a distinct family, suggesting that risk variants localize to specific transcription factor targeted OCRs. We find an enrichment of PD risk variants in OCRs targeted by bHLH transcription factors. There is a high degree of similarity between recognition motifs of members of the large bHLH transcription factor family, which provides several binding candidates. bHLH transcription factors are key determinants of neural cell fate specification and differentiation^34^. Many of the transcription factors that are candidates to target this subset of open chromatin are mainly expressed and function in the developing nervous system, and thus more likely to be involved in neurodevelopmental diseases. However, a developmental component to PD pathogenesis cannot be excluded, conceivably laying the groundworks for the brain’s future vulnerability to or resilience against adult onset neurodegeneration^34^. Some bHLH transcription factors also function in adult neurons, such as transcription factor 4 (TCF4), which is the second best match to the de novo motif identified by HOMER^40^. Autosomal dominant mutations and deletions in TCF4 cause the neurodevelopmental disorder Pitt-Hopkins syndrome, while common variants at the TCF4 locus are associated with schizophrenia risk^41–44^.

Epigenomic studies of the brain have predominantly been conducted in bulk tissue, which may perturb the detection of cell type specific regulatory elements due to measurement of an average signal across a heterogeneous population of cells. In contrast, Fullard et al. applied ATAC-seq to sorted nuclei^20^. This enables the distinction between OCRs in neurons vs non-neuronal cells, which we consider to be a major strength of this dataset.

We draw our conclusions based on results from two different enrichment tests. Due to the overlap between OCRs in the different cell types and motif-subsets, adjustment for multiple testing by Bonferroni correction may be considered to be a very strict significance threshold potentially leading to false negatives. This is mostly relevant to GoShifter, which is reported to have very conservative estimates^45^. It should however be noted that it is only the motif-containing OCR subset passing our set significance threshold which is also significant in analyses based on the alternative de novo motif discovery method. We consider it a strength of our study that we employ two different methods for de novo motif discovery. HOMER and MEME-ChIP are widely used tools for motif analysis of large DNA sequence data sets. The analyses are performed in parallel and both show an enrichment of PD risk variants in OCRs targeted by bHLH transcription factors, thus increasing the robustness of this finding.

We analyse two non-brain related disorders as negative controls and find no evidence of enrichment in cortical neurons, showing some degree of specificity of our findings to PD. In further interpretations of our results it is important to recognize that the detection of motifs and potential binding of bHLH transcription factors could be a marker of an active regulatory region also bound by other regulatory factors, of which one exerts the true causal effect on PD risk. We cannot exclude the possibility that an observed enrichment is due to unaccounted colocalization with other annotations. This limits the inference of causality and must be taken into account when interpreting results from enrichment analysis.

In our study, integration of GWAS signals with sites of open chromatin suggests that neurons in the superior temporal cortex and additional cortical regions mediate genetic risk for PD. Motif analysis performed in neurons of the superior temporal cortex shows that PD risk variants significantly overlap OCRs targeted by members of the bHLH transcription factor family, pointing to an involvement of these transcriptional networks in PD risk mechanisms. Additional investigations are needed to further explore the role of bHLH transcription factors in PD. Our study also demonstrates that ATAC-seq data coupled with motif analysis may be used in the assessment of hundreds of different transcription factors in a relevant cellular context, something that is not possible with existing transcription factor ChIP-seq data. Future studies addressing regulatory mechanisms in PD will benefit from improved computational approaches to predict transcription factor binding sites as a complement to ChIP-seq. Novel computational methods highlight the importance of both motif-based and chromatin accessibility features as pivotal to yield high performance predictions for most transcription factors^46,47^. Generation of epigenomic data with increased cellular resolution in brain related cell types would thus provide another valuable resource to study the involvement of transcription factors in neurodegenerative diseases.

## Supplementary Information

Supplementary Information 1.

Supplementary Information 2.

Supplementary Information 3.

Supplementary Information 4.

Supplementary Information 5.

Supplementary Information 6.

## Data Availability

The datasets analysed during the current study are available from Brain Open Chromatin Atlas (BOCA) (https://bendlj01.u.hpc.mssm.edu/multireg/resources/boca_peaks.zip) and UCSC Genome Browser (http://hgdownload.soe.ucsc.edu/goldenPath/hg19/bigZips/latest/hg19.fa.masked.gz).
