# Supplementary material for: Integrative analysis identifies bHLH transcription factors as contributors to Parkinson’s disease risk mechanisms
Source: Sci Rep. 2021 Feb 10;11:3502. doi: 10.1038/s41598-021-83087-2 (PMC7875985; doi:10.1038/s41598-021-83087-2)
Supplement: Supplementary file 1 — Supplementary Information 1. [file 41598_2021_83087_MOESM1_ESM.pdf]

Supplementary Information

Integrative analysis identifies bHLH transcription factors as contributors to Parkinson's disease risk mechanisms

Victoria Berge-Seidl[1,2], Lasse Pihlstrøm[1], Mathias Toft[1,2*]

[1]Department of Neurology, Oslo University Hospital, Oslo, Norway

[2]Faculty of Medicine, University of Oslo, Oslo, Norway

* Corresponding author

<u>This file includes:</u>
Captions for Supplementary Table S1 – S9
Supplementary Table S2, S4, S7 and S8
Supplementary Figure S1 – S3

Supplementary Table S1. List of variants in high linkage disequilibrium with index variants representing independent genome-wide significant association signals in PD, IBD and PEF. For PD, all index variants passed analysis with the webserver Snipa with the exception of rs34637584 and rs76763715 due to not being in the reference set or population. The IBD index variants rs75900472 and rs144344067, and the PEF index variants rs9274247 and rs79412431 did not pass analysis for the same reason. QRSID is the query variant and defines the index variant, while RSID defines a proxy variant in high linkage disequilibrium ($r^2 > 0.8$) with the index variant. POS1 refers to the index variant position and POS2 is the proxy variant position. The degree of linkage disequilibrium between QRSID and RSID is provided as R-squared (R2) and DPRIME. PD, Parkinson's disease; IBD, Inflammatory bowel disease; PEF, Peak expiratory flow; MAF, Minor allele frequency.

Supplementary Table S2. Results from enrichment analysis of negative controls within open chromatin regions in brain neurons.

	PEF		IBD		
Cell type	GoShifter adj. p-val (p-val)	GREGOR adj. p-val (p-val)	GoShifter adj. p-val (p-val)	GREGOR adj. p-val (p-val)	No. ATAC-seq peaks
STC	1 (0.314)	0.93 (0.066)	1 (0.46)	1 (0.319)	76145
VLPFC	1 (0.424)	0.696 (0.05)	1 (0.627)	1 (0.481)	86082
ITC	1 (0.557)	1 (0.171)	1 (0.699)	1 (0.816)	65346
PMC	1 (0.482)	1 (0.074)	1 (0.949)	1 (0.9)	84995
ACC	1 (0.811)	1 (0.291)	1 (0.789)	1 (0.677)	70654
OFC	1 (0.666)	1 (0.184)	1 (0.866)	1 (0.649)	81621
INS	1 (0.747)	1 (0.306)	1 (0.366)	1 (0.406)	68261
DLPFC	1 (0.515)	1 (0.117)	1 (0.344)	1 (0.232)	74825
PVC	1 (0.215)	1 (0.099)	1 (0.722)	1 (0.637)	51874
NAC	1 (0.884)	1 (0.71)	1 (0.981)	1 (0.83)	77290
MDT	1 (0.193)	1 (0.169)	1 (0.828)	1 (0.679)	69913
HIPP	1 (0.616)	1 (0.282)	1 (0.797)	1 (0.705)	80571
AMY	1 (0.718)	1 (0.529)	1 (0.681)	1 (0.449)	38564
PUT	1 (0.874)	1 (0.419)	1 (0.959)	1 (0.577)	100752

There are no adj. p-val < 0.05 in any of the tested cell types. We adjusted for multiple testing by Bonferroni correction, adjusting for 14 tests. Unadjusted p-values are provided in parenthesis. No. ATAC-seq peaks refers to the total number of peaks, representing open chromatin regions, in the analysed cell types. PEF, Peak expiratory flow; IBD, Inflammatory bowel disease; ACC, Anterior cingulate cortex; AMY, Amygdala; DLPFC, Dorsolateral prefrontal cortex; HIPP, Hippocampus; INS, Insula; ITC, Inferior temporal cortex; MDT, Mediodorsal thalamus; NAC, Nucleus Accumbens; OFC, Orbitofrontal cortex; PMC, Primary motor cortex; PUT, Putamen; PVC, Primary visual cortex; STC, Superior temporal cortex; VLPFC, Ventrolateral prefrontal cortex.

Supplementary Table S3. Results from *de novo* motif analysis of open chromatin regions in superior temporal cortex neurons performed with HOMER

Supplementary Table S4. Results from enrichment analysis of negative controls within motif-containing open chromatin region sets identified with HOMER.

	PEF		IBD		
Motif-containing OCR sets	GoShifter adj. p-val (p-val)	GREGOR adj. p-val (p-val)	GoShifter adj. p-val (p-val)	GREGOR adj. p-val (p-val)	No. ATAC-seq peaks
Olig2*	1 (0.214)	1 (0.063)	1 (0.692)	1 (0.472)	21924
POL010.1_DCE	1 (0.293)	1 (0.089)	1 (0.571)	1 (0.507)	37574
NRF1	1 (0.436)	1 (0.663)	1 (0.820)	1 (0.563)	7729
NFIA	1 (0.510)	1 (0.178)	1 (0.371)	1 (0.309)	37903
Sp2	1 (0.861)	1 (0.972)	1 (0.918)	1 (0.592)	7566
Egr2	1 (0.223)	1 (0.148)	1 (0.926)	1 (0.682)	25202
NFY	1 (0.657)	1 (0.893)	1 (0.598)	1 (0.722)	5806
PB0080.1_Tbp_1	1 (0.302)	1 (0.109)	1 (1)	1 (1)	5118
ETV2	1 (0.252)	1 (0.235)	1 (0.734)	1 (0.369)	10961
CTCF	1 (0.817)	1 (0.338)	1 (0.258)	1 (0.161)	6257
Mef2c	1 (0.237)	0.239 (0.010)	1 (0.942)	1 (0.722)	17566
PB0013.1_Eomes_1	1 (0.756)	1 (0.448)	1 (0.330)	1 (0.221)	29438
Atf1	1 (0.653)	1 (0.645)	1 (0.341)	1 (0.180)	5809
BORIS	1 (0.874)	1 (0.444)	1 (0.371)	1 (0.623)	6018
POL002.1_INR	1 (0.408)	1 (0.161)	1 (0.884)	1 (0.959)	34917
SPDEF	1 (0.976)	1 (0.883)	1 (0.748)	1 (0.383)	18884
MafF	1 (0.967)	1 (0.840)	1 (0.674)	1 (0.495)	34091
GFY	1 (0.261)	1 (0.415)	1 (1)	1 (1)	1196
Rfx5	1 (0.300)	0.901 (0.039)	1 (0.725)	1 (0.522)	7410
Fra1	1 (0.592)	1 (0.119)	1 (0.665)	1 (0.296)	9557
NFIL3	1 (0.628)	1 (0.581)	1 (1)	1 (1)	4431
Rfx1	1 (0.841)	1 (0.518)	1 (1)	1 (1)	3337
noMotif	1 (1)	1 (1)	1 (1)	1 (1)	1196

There are no adj. p-val < 0.05 in any of the motif-containing OCR sets. We adjusted for multiple testing by Bonferroni correction, adjusting for 23 tests. Unadjusted p-values are provided in parenthesis. No. ATAC-seq peaks refers to the total number of peaks, representing OCRs, in the analysed motif-containing OCR sets. The total number of ATAC-seq peaks in superior temporal cortex neurons is 76145. PEF, Peak expiratory flow; IBD, Inflammatory bowel disease; OCR, Open chromatin region.

Supplementary Table S5. The 25 most significant results from *de novo* motif analysis of open chromatin regions in superior temporal cortex neurons performed with MEME-ChIP. The three most similar known motifs are listed. Only known motifs with a TOMTOM similarity E-value of less than 1.0 to the discovered motif are shown.

Supplementary Table S6. Known motifs matched to *de novo* motifs identified with HOMER and MEME-ChIP. Known motifs with a similarity score of 0.70 and higher to the 22 *de novo* motifs discovered with HOMER, known motifs with TOMTOM similarity E-value of less than 1.0 to the 25 most significant *de novo* motifs discovered with MEME-ChIP, and known motifs matched to both HOMER and MEME-ChIP *de novo* motifs are listed. Known motifs matched to *de novo* motifs identified with HOMER are either from Jaspar motif database (J), Homer motif database (H), or from both. All known motifs matched to *de novo* motifs identified with MEME-ChIP are from Jaspar motif database. A known motif may be matched to more than one *de novo* motif, but is only listed once.

Supplementary Table S7. Results from enrichment analysis of PD risk variants and negative controls in open chromatin region sets containing *de novo* motifs identified with MEME-ChIP.

	**PD**		**PEF**		**IBD**		
Motif – containing OCR sets	GoShifter adj. p-val (p-val)	GREGOR adj. p-val (p-val)	GoShifter adj. p-val (p-val)	GREGOR adj. p-val (p-val)	GoShifter adj. p-val (p-val)	GREGOR adj. p-val (p-val)	No. ATAC-seq peaks
SP1	0.403 (0.031)	**4.07×10^{-03} (3.13×10^{-04})**	1 (0.693)	1 (0.793)	1 (0.917)	1 (0.656)	11838
ZNF263	0.579 (0.045)	**1.69×10^{-03} (1.30×10^{-04})**	1 (0.838)	1 (0.724)	1 (0.986)	1 (0.868)	17280
RBPJ	1 (0.082)	1 (0.088)	1 (0.954)	1 (0.927)	1 (0.602)	1 (0.651)	8710
NEUROD1_2	1 (0.090)	0.097 (7.50×10^{-03})	1 (0.760)	1 (0.480)	1 (0.928)	1 (0.614)	9948
SPIB	1 (0.110)	0.673 (0.052)	1 (0.829)	1 (0.324)	1 (0.361)	1 (0.238)	12998
TBP	1 (0.263)	1 (0.333)	1 (0.511)	1 (0.121)	1 (0.347)	1 (0.222)	10679
KLF9	1 (0.425)	0.323 (0.025)	1 (0.901)	1 (0.856)	1 (0.976)	1 (0.945)	8071
NEUROD1_1	1 (0.548)	1 (0.469)	0.689 (0.053)	0.793 (0.061)	1 (1)	1 (1)	6772
NRF1	1 (0.589)	1 (0.103)	1 (1)	1 (1)	1 (0.873)	1 (0.656)	3538
NHLH1	1 (0.620)	1 (0.225)	1 (0.490)	1 (0.470)	1 (0.833)	1 (0.501)	9684
FOSL2	1 (0.640)	1 (0.170)	1 (0.568)	1 (0.087)	1 (0.650)	1 (0.321)	9021
MEF2C	1 (0.647)	1 (0.945)	1 (0.458)	1 (0.116)	1 (0.305)	1 (0.367)	11541
ZNF384	1 (0.726)	1 (0.195)	1 (0.802)	1 (0.316)	1 (0.220)	1 (0.501)	16464

The motif-containing OCR sets are named after the best matched known motif. We adjusted for multiple testing by Bonferroni correction, adjusting for 13 tests. Unadjusted p-values are provided in parenthesis. Adjusted p-val < 0.05 are written in bold. No. ATAC-seq peaks refers to the total number of peaks, representing OCRs, in the analysed motif-containing OCR sets. The total number of ATAC-seq peaks in superior temporal cortex neurons is 76145. PD, Parkinson’s disease; PEF, Peak expiratory flow; IBD, Inflammatory bowel disease; OCR, Open chromatin region.

Supplementary Table S8. Results from enrichment analysis of negative controls within open chromatin region sets containing known motifs identified with MEME-ChIP.

	**PEF**		**IBD**		
Motif-containing OCR sets	GoShifter adj. p-val (p-val)	GREGOR adj. p-val (p-val)	GoShifter adj. p-val (p-val)	GREGOR adj. p-val (p-val)	No. ATAC-seq peaks
NEUROD1	1 (0.244)	1 (0.359)	1 (0.992)	1 (0.977)	12197
SP1	1 (0.691)	1 (0.867)	1 (0.900)	1 (0.553)	19373
ZNF263	1 (0.059)	0.256 (0.014)	1 (0.560)	1 (0.257)	27496
NHLH1	1 (0.925)	1 (0.904)	1 (0.862)	1 (0.671)	8288
TEAD2	1 (0.927)	1 (0.455)	1 (0.971)	1 (0.899)	7000
RBPJ	1 (0.846)	1 (0.578)	1 (0.351)	1 (0.296)	11637
KLF9	1 (0.164)	1 (0.241)	1 (0.962)	1 (0.869)	12364
NRF1	1 (0.742)	1 (0.852)	1 (0.688)	1 (0.142)	7740
SPIC	1 (1)	1 (1)	1 (0.234)	1 (0.126)	8737
SPIB	1 (1)	1 (1)	1 (0.603)	1 (0.433)	9300
ZIC1	1 (0.675)	1 (0.707)	1 (0.790)	1 (0.784)	6952
Stat5a::Stat5b	1 (0.540)	1 (0.457)	1 (0.561)	1 (0.648)	9783
ZNF384	1 (0.500)	1 (0.172)	1 (0.478)	1 (0.549)	14373
MEF2C	1 (0.432)	0.555 (0.031)	1 (0.974)	1 (0.901)	16923
FOSL2	1 (0.392)	1 (0.122)	1 (0.655)	1 (0.356)	9141
FOXP1	1 (0.907)	1 (0.801)	1 (0.681)	1 (0.485)	6847
TBP	1 (0.433)	1 (0.103)	1 (0.562)	1 (0.410)	5011
CREB1	1 (0.546)	1 (0.174)	1 (0.463)	1 (0.171)	2994

There are no adj. p-val < 0.05 in any of the motif-containing OCR sets. We adjusted for multiple testing by Bonferroni correction, adjusting for 18 tests. Unadjusted p-values are provided in parenthesis. No. ATAC-seq peaks refers to the total number of peaks, representing OCRs, in the analysed motif-containing OCR sets. The total number of ATAC-seq peaks in superior temporal cortex neurons is 76145. PEF, Peak expiratory flow; IBD, Inflammatory bowel disease; OCR, Open chromatin region.

Supplementary Table S9. PD association signals and proxy variants that overlap open chromatin region subsets targeted by bHLH transcription factors. PD association signals (represented by the top-hit variant) and proxy variants that overlap the OCR subset containing the HOMER *de novo* motif best matched to Olig2 and the OCR subset containing the NEUROD1 motif identified by MEME-ChIP are listed. The overlap has been identified by analysis with GoShifter. PD association signals and proxy variants that overlap both the Olig2 OCR subset and the NEUROD1 OCR subset are written in bold. PD, Parkinson's disease; bHLH, Basic Helix-Loop-Helix; OCR, Open chromatin region.

Supplementary Figure S1. Heatmap of pairwise intersections of Jaccard statistic of open chromatin regions in brain.

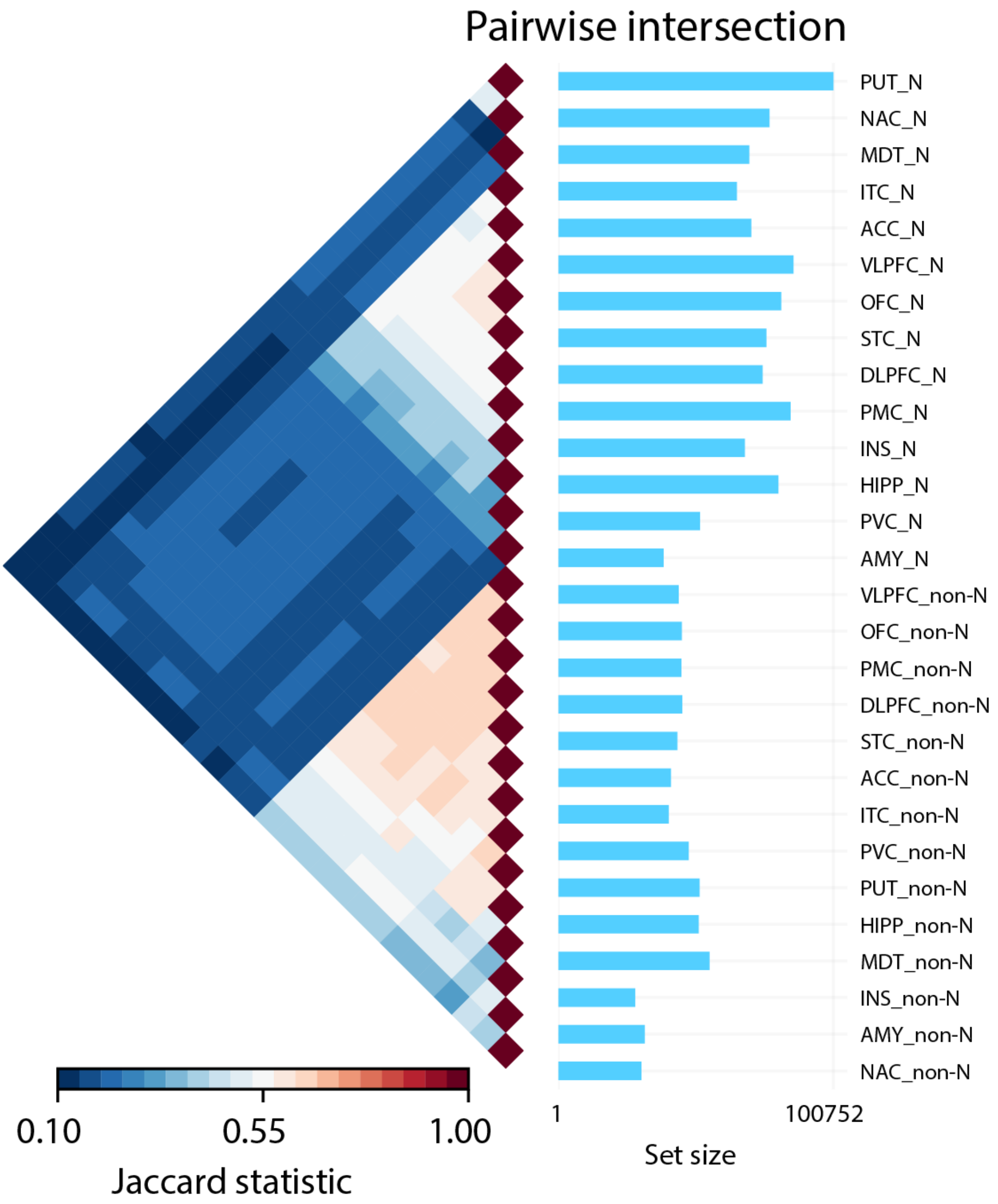

The set size refers to the number of open chromatin regions in each dataset. N, Neuronal; non-N, non-Neuronal; ACC, Anterior cingulate cortex; AMY, Amygdala; DLPFC, Dorsolateral prefrontal cortex; HIPP, Hippocampus; INS, Insula; ITC, Inferior temporal cortex; MDT, Mediodorsal thalamus; NAC, Nucleus Accumbens; OFC, Orbitofrontal cortex; PMC, Primary motor cortex; PUT, Putamen; PVC, Primary visual cortex; STC, Superior temporal cortex; VLPFC, Ventrolateral prefrontal cortex.

Supplementary Figure S2. Known motifs matched to the HOMER *de novo* motif located in the open chromatin region subset enriched with Parkinson's disease risk variants

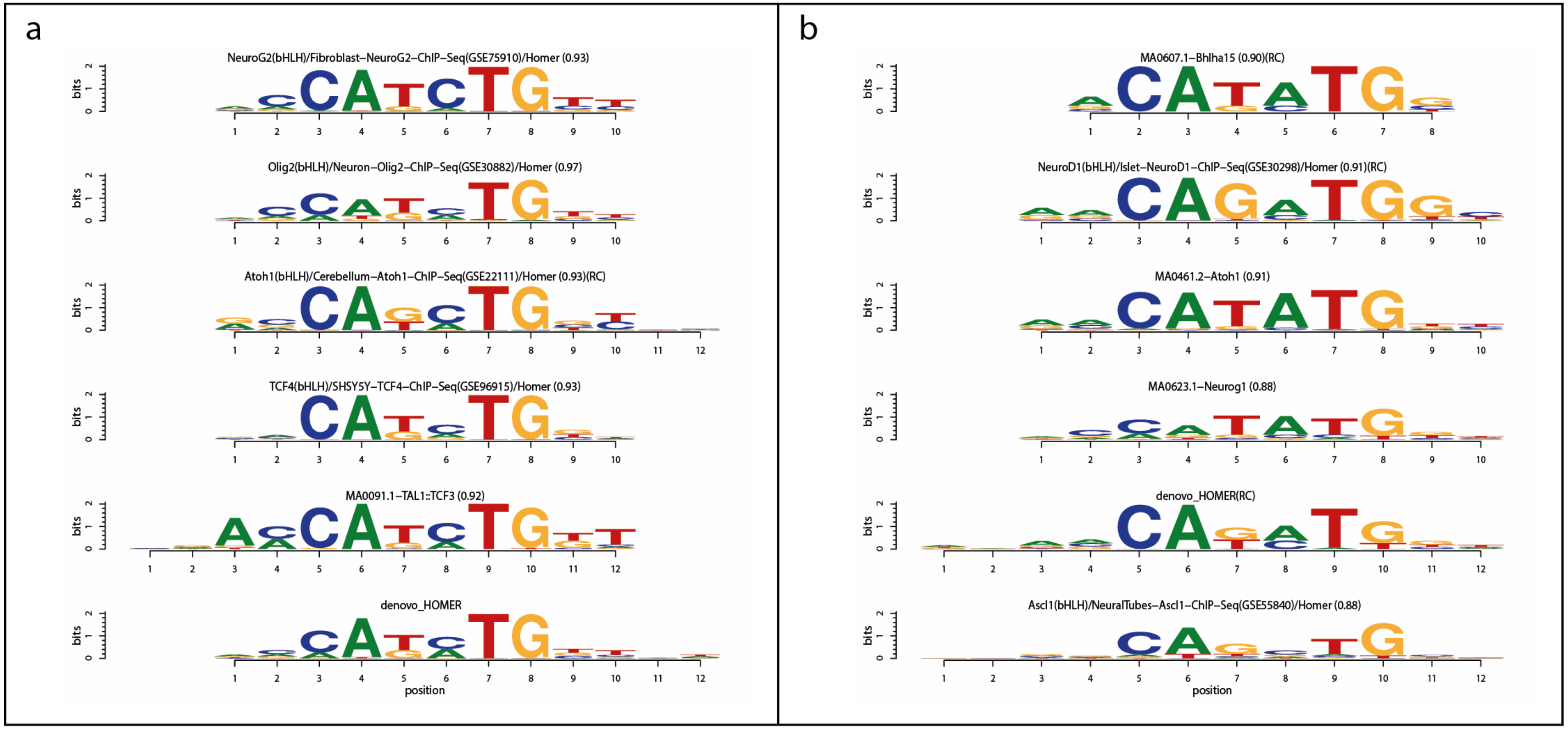

a) *De novo* motif compared to the five known motifs with highest similarity scores. b) *De novo* motif compared to known motifs with 6th – 10th highest similarity scores. The similarity score is provided in parenthesis after the motif name. RC, Reverse complement.

Supplementary Figure S3. Overlap between PD risk variants that locate to the two enriched motif-containing open chromatin region subsets

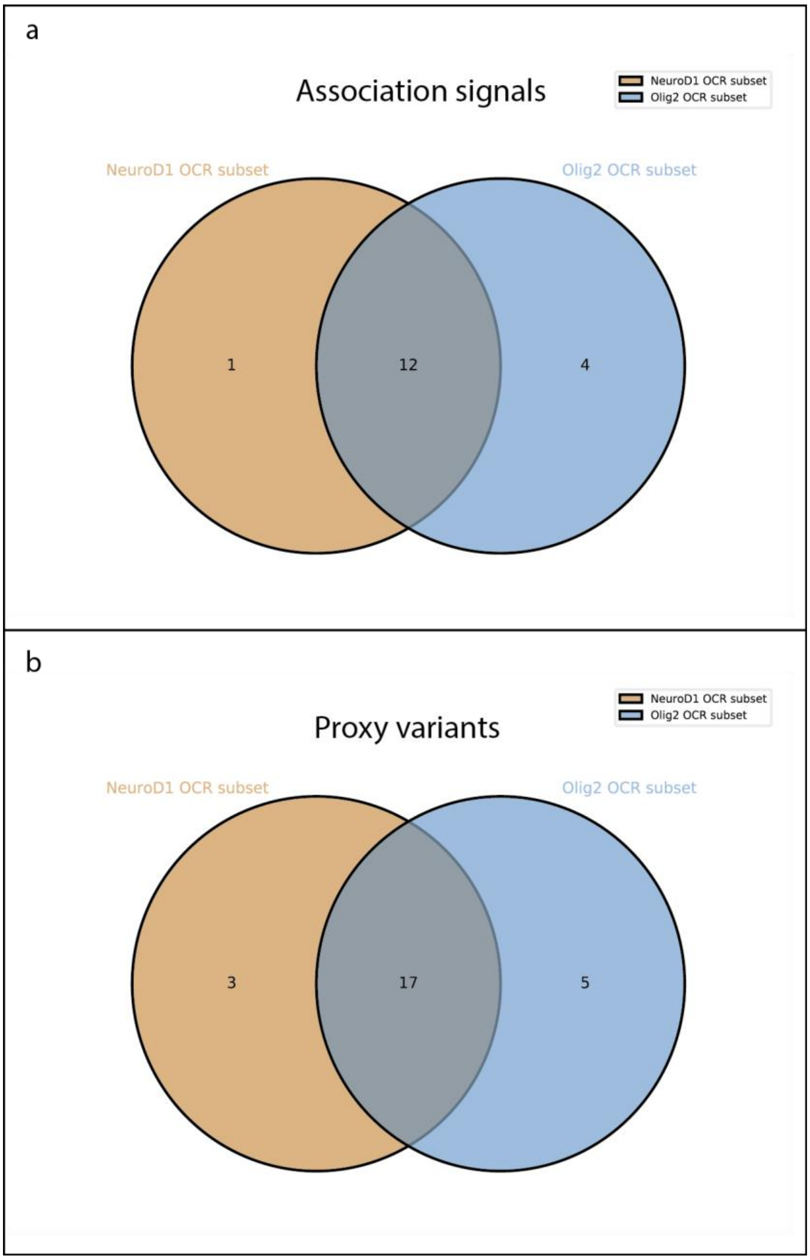

a) Venn diagram illustrating the overlap between PD association signals that locate to the NEUROD1 OCR subset and the Olig2 OCR subset, showing that 12 association signals locate to both subsets. b) Venn diagram illustrating the overlap between proxy variants that locate to the NEUROD1 OCR subset and the Olig2 OCR subset, showing that 17 proxy variants locate to both subsets. Venn diagrams have been created with the software Intervene. PD, Parkinson’s disease; OCR, Open chromatin region.
